# Functional analysis of photosynthetic pigment binding complexes in the green alga *Haematococcus pluvialis* reveals distribution of astaxanthin in Photosystems

**DOI:** 10.1038/s41598-017-16641-6

**Published:** 2017-11-24

**Authors:** Francesco Mascia, Laura Girolomoni, Marcelo J. P. Alcocer, Ilaria Bargigia, Federico Perozeni, Stefano Cazzaniga, Giulio Cerullo, Cosimo D’Andrea, Matteo Ballottari

**Affiliations:** 1 Dipartimento di Biotecnologie, Università di Verona, Strada Le Grazie 15, I-37134 Verona, Italy; 2 Center for Nano Science and Technology @PoliMi, Istituto Italiano di Tecnologia, via Pascoli 70/3, 20133 Milano, Italy; 3 IFN-CNR, Department of Physics, Politecnico di Milano, P.za L. da Vinci 32, 20133 Milano, Italy

## Abstract

Astaxanthin is a ketocarotenoid produced by photosynthetic microalgae. It is a pigment of high industrial interest in acquaculture, cosmetics, and nutraceutics due to its strong antioxidant power. *Haematococcus pluvialis*, a fresh-water microalga, accumulates high levels of astaxanthin upon oxidative stress, reaching values up to 5% per dry weight. *H. pluvialis* accumulates astaxanthin in oil droplets in the cytoplasm, while the chloroplast volume is reduced. In this work, we investigate the biochemical and spectroscopic properties of the *H. pluvialis* pigment binding complexes responsible for light harvesting and energy conversion. Our findings demonstrate that the main features of chlorophyll and carotenoid binding complexes previously reported for higher plants or *Chlamydomonas reinhardtii* are preserved under control conditions. Transition to astaxanthin rich cysts however leads to destabilization of the Photosystems. Surprisingly, astaxanthin was found to be bound to both Photosystem I and II, partially substituting β-carotene, and thus demonstrating possible astaxanthin biosynthesis in the plastids or transport from the cytoplasm to the chloroplast. Astaxanthin binding to Photosystems does not however improve their photoprotection, but rather reduces the efficiency of excitation energy transfer to the reaction centers. We thus propose that astaxanthin binding partially destabilizes Photosystem I and II.

## Introduction

*Haematococcus pluvialis* is a photosynthetic fresh-water microalga which accumulates a high level of the ketocarotenoid astaxanthin (up to 5% DW)^1–4^. Astaxanthin is mainly used as coloring agent in aquaculture but it has been also reported to be a strong antioxidant, preventing production of reactive oxygen species (ROS) and lipid peroxidation in solution and in several biologic systems^5–9^. Numerous studies have shown that astaxanthin has health-promoting effects in the prevention and treatment of various diseases such as cancers, chronic inflammations, metabolic syndrome, cardiovascular and gastrointestinal diseases, as well as enhancing the immune system and protecting the skin from radiation injury^10^. Astaxanthin cannot be manufactured in animals and therefore must be consumed in the diet. This carotenoid (Car) is thus of great interest for several industrial sectors and has a high market potential. Many studies have addressed the role of astaxanthin in *H. pluvialis* and the phenotypical characterization of this alga^1–3,11–16^. The lifecycle of *H. pluvialis* includes four phases and astaxanthin is accumulated only in the aplanospores phase, which is induced under stress conditions such as high light intensity, nutrient starvation, high salinity or low/high temperatures^1,12,17,18^. Astaxanthin production from *H. pluvialis* mass cultivation is commonly carried out in a two-stage batch culture; biomass production occurs in the first stage (green stage), while in the second stage (red stage) the cultures are stressed to induce astaxanthin accumulation^19^. Astaxanthin is accumulated mainly at the level of the endoplasmatic reticulum in the form of mono- and di-esters by using β-carotene as precursor^20,21^. While several reports focused on astaxanthin production and its application for humans as a nutraceutic, details regarding the role of this Car in *H. pluvialis* cells are still not complete^1,4,12,15,16,22–24^. The astaxanthin biosynthetic pathway depends on carbon fixation by the photosynthetic process in the chloroplasts. During transition to astaxanthin rich cysts, Car biosynthesis is triggered and the plastids are degraded^25^. Photosynthetic processes are functionally divided in two phases; the light phase and dark phase. The light phase takes place in the thylakoid membranes, with light energy being harvested and converted into chemical energy in the form of NADPH and ATP. The subsequent dark phase is where NADPH and ATP are used in the stroma by Calvin-Benson cycle for enzymatic CO_2_ fixation and reduction to carbohydrates. PSII and PSI are responsible for energy conversion, cytochrome b_6/f_ contributes to electron transport and proton translocation in the lumen, and ATPase catalyzes ATP synthesis using the energy derived from the transmembrane proton gradient. PSI and PSII are pigment binding proteins composed of a *core* complex and antenna proteins called Light Harvesting Complexes (LHC)^26–29^. The *core* complex binds chlorophyll (Chl) *a* and β-carotene, whilst the antenna proteins bind Chl *a*, Chl *b*, and xanthophylls^26,30^. Pigments bound by the photosystems absorb photons and transfer excitation energy to the reaction centers. In particular PSI was observed to trap excitation energy at the reaction center (RC) faster than PSII^31^. Higher plants and unicellular microalgae show some differences in both PSII and PSI supramolecular organization, with different stoichiometries of LHC proteins per PSI(II) core complexes. Four LHC subunits, called Lhca1-4, were found bound to PSI core complex of *A. thaliana*, while 7 to 9 different Lhca complexes were identified in the PSI-LHCI complexes from the green alga *Chlamydomonas reinhardtii*. In the case of PSII, the number of LHC complexes bound, called Lhcb, is more variable and depends on growth conditions^28–30,32,33^. Very little information is available regarding the photosynthetic complexes of the green alga *H. pluvialis* and how they are modulated during cyst formation and astaxanthin accumulation. Moreover, it is still under debate if astaxanthin accumulation has some photoprotective function at the level of the chloroplast^12,22,34,35^. The aim of this work is to characterize the photosynthetic complexes in *H. pluvialis* and the possible role of astaxanthin in the photosynthetic apparatus during acclimation to high light and transition to the red stage.

## Results

### Astaxanthin accumulation in ***H. pluvialis***

**Figure 1:**
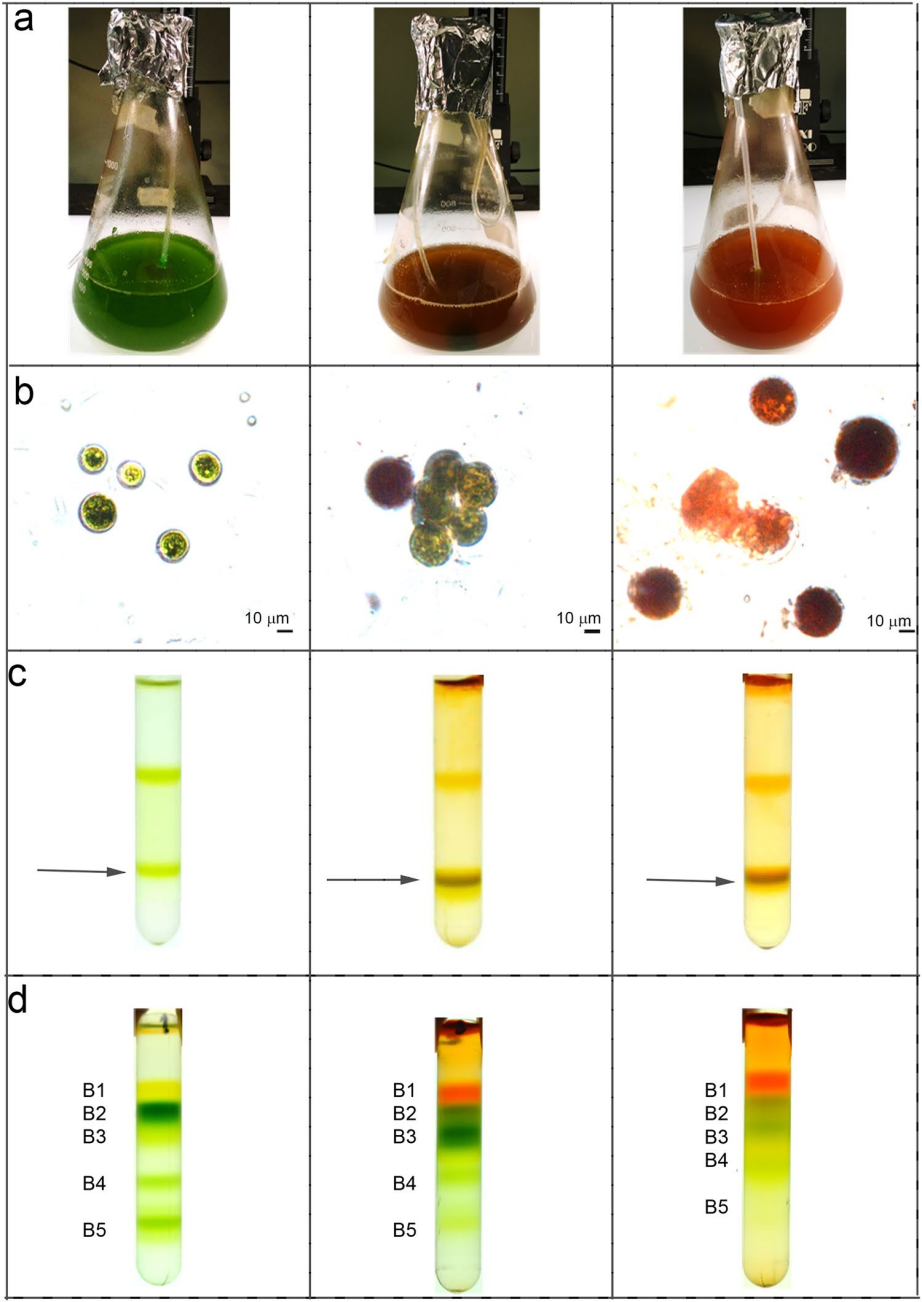
Cell cultivation, membranes and pigment binding complexes isolation. Panel a: *H. pluvialis* cultures grown under 3 different stress conditions. Green: 50 µmol m^−2^s^−1^ for 7 days; Orange: 400 µmol m^−2^s^−1^ for 3 days; Red: 400 µmol m^−2^s^−1^ for 6 days. Panel b: microscope observation of cells grown as in Panel A. Panel c: isolation of plastid membranes from G, O and R cells. Purified membranes are indicated by the arrow. Panel d: Sucrose gradient ultracentrifugation separation of pigment binding complexes from plastid membranes solubilized in β-DM 1%.

**Figure 2:**
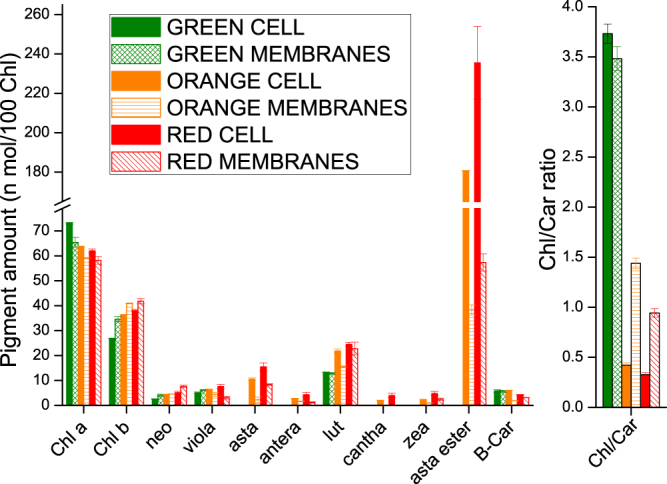
Pigment analysis on *H. pluvialis* whole cells and isolated membranes. Pigment extracts were analyzed by HPLC. Pigment data were normalized to 100 chlorophylls. Chl: chlorophyll; Car: carotenoid; Neo: neoxanthin; Viola: violaxanthin; Lute: Lutein; Antera: anteraxanthin; Cantha: canthaxanthin; Zea: zeaxanthin; B-Car: β-carotene; Asta: astaxanthin; Asta ester: esterified forms of astaxanthin. Standard deviation (s.d.) are reported (n = 3).

*Haematococcus pluvialis* cells were grown in BG11 medium at 50 µE for 7 days (hereafter, referred as G/Green), at 400 µE for 3 days (hereafter, referred as O/Orange) and at 400 µE for 6 days (hereafter, referred as R/Red). As reported in Fig. 1, a clear change in the culture color appeared under the three different growth conditions, from green, to brownish, to red for G, O and R conditions respectively. *H. pluvialis* cells grown were then observed in bright-field microscopy (Fig. 1b). Cells grown in the G condition were round green cells with a distinct cell wall layer; in the O condition, cells became reddish, likely due to astaxanthin accumulation, but green chloroplasts were still visible; some cells in O and all cells R were characterized by a complete transition into a red stage, with strong astaxanthin accumulation. In this condition, partial cell degradation was also evident. Pigment composition was investigated by HPLC and reported in Fig. 2. A strong reduction of chlorophyll to carotenoid (Chl/Car) ratio was observed in O and R cells with the higher Car accumulation in R cells. Astaxanthin esters were the predominant Car species found in O and R cells, while no traces of astaxanthin were found in G cells. Traces of canthaxanthin, a precursor of astaxanthin, were also found in O and R cells. Incidentally finding of Chls, β-carotene and xanthophylls in O and R cells suggested the residual presence of photosynthetic complexes, responsible for the photosynthetic activity previously reported for *H. pluvialis* during transition to the red stage^12^. Lutein and neoxanthin were more abundant on a Chl basis in O and R conditions, while beta-carotene was reduced in R cells. Since lutein and neoxanthin are bound only to LHC complexes, while beta-carotene is essentially bound only to PSI or PSII core subunits, the increased neoxanthin or lutein to beta carotene ratios imply a partial degradation of core subunits during high light exposure.

### Isolation and characterization of pigment binding complexes of *H. pluvialis* in different growth conditions

**Figure 3:**
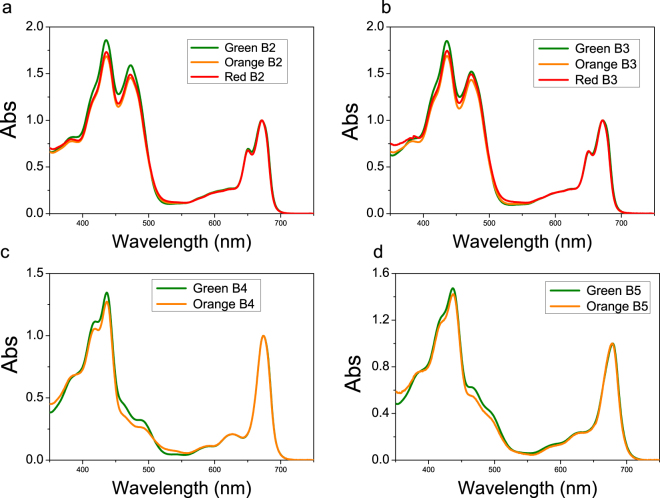
Absorption spectra of pigment binding complexes isolated from *H. pluvialis*. Absorption spectra of B2, B3, B4 and B5 fraction (Fig. 1d) were normalized to the maximum absorption peak in the 600 nm and 700 nm region.

**Figure 4:**
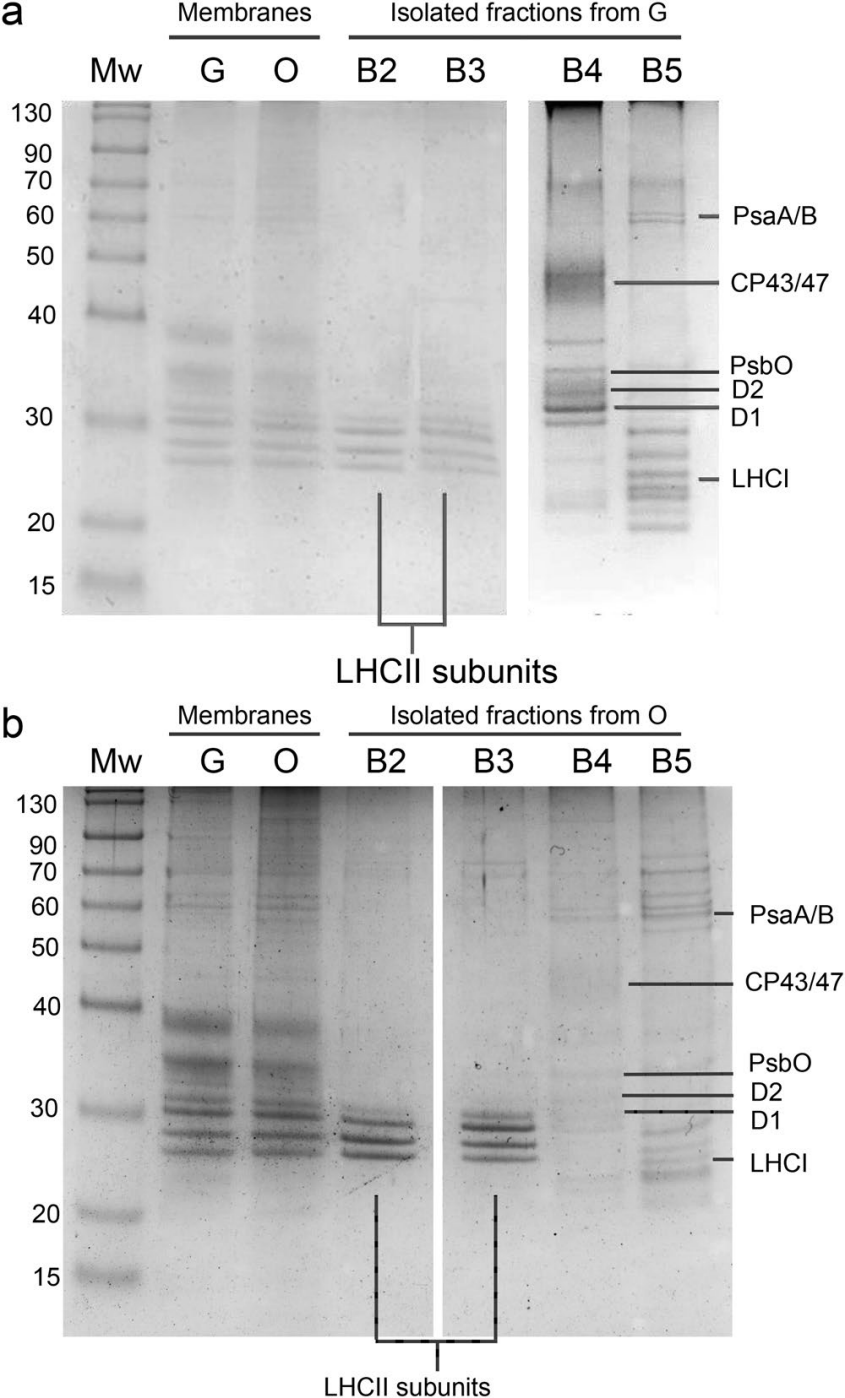
SDS-PAGE of *H. pluvialis* purified membranes and isolated pigment binding complexes. Panel a: Isolated B2-5 fractions from Green cells (Fig. 1) samples; Panel b: Isolated B2-5 fraction from Orange cells. SDS-PAGE gels were Coomassie stained. Lanes loaded in separate gels are divided by white spaces. Mw: molecular weight marker; G: proteins from “Green” cells grown at 50 μE; O: proteins from “Orange” cells grown at 400 μE for 3 days.

Thylakoid membranes of G, O and R cells were isolated by mechanical cell disruption followed by selective centrifugations with a final purification step by ultracentrifugation in a sucrose step gradient. As reported in Fig. 1c, membranes at similar sucrose densities were recovered from both G, O and R samples. Pigment composition of the purified membranes was then investigated by HPLC and reported in Fig. 2. In O and R samples, the Chl/Car ratio was significantly reduced in purified thylakoid membranes as compared to whole cells. The observed reduction is mainly related to a strong decrease of astaxanthin, either in free or esterified forms, as compared to pigment extracts from whole cells. A reduction of β-carotene, anteraxanthin, zeaxanthin, canthaxanthin and lutein was also observed in O and R membranes as compared to whole cells, even if it was less pronounced when compared to astaxanthin. Since it has been reported that astaxanthin is accumulated only outside the plastids in *H. pluvialis* ^20,21,25^, the results obtained could be due to a co-purification of thylakoid membranes and astaxanthin rich oil droplets of similar densities. Since the presence of astaxanthin in thylakoid membranes has been reported for transgenic plants accumulating this Car^36–38^, the possible presence of astaxanthin molecules bound to pigment binding complexes was investigated using treated membranes. Purified membranes were solubilized and the Chl binding complexes were isolated by ultracentrifugation in a sucrose gradient^39^. This ultracentrifugation step allowed a separation of the different photosynthetic complexes, based on their molecular density. Five bands (B1, B2, B3, B4, B5) were observed in every condition (G, O, R) (Fig. 1d). B1-5 fractions were recovered and their absorption spectra investigated. The B1 band was composed of free pigments, as indicated by the Chl Qy absorption peak below 670 nm (Supplemental data Figure S1). Absorption spectra of B2 and B3 fractions (Fig. 3a,b) were similar in G, O and R conditions, resembling the features of LHC antenna proteins with two peaks in the Qy region attributable to Chl *a* (672 nm) and *b* (650 nm). Based on their molecular density, B2 and B3 were composed of monomeric and trimeric LHC proteins respectively. B4 spectra showed an almost complete absence of the 470 nm and 650 nm peaks (Fig. 3c), which are both related to Chl *b*. This indicates a high Chl *a*/*b* ratio, and hints at the presence of PSII-core in this fraction. Comparing O B4 with G B4, it is possible to notice a decreased absorbance at ~470 nm coupled with an increased absorbance at ~530 nm, which suggests the presence of astaxanthin. B5 spectra (Fig. 3d) reveal the predominance of Chl a with a maximum absorbance in the Qy region at 679 nm, although Chl *b* contributions at 650 and 470 nm were still present in a lower amount. B5 was thus likely composed of the PSI-LHCI supercomplex. PSII-core (B4) and PSI (B5) fractions from R were not harvested since the bands in sucrose gradient were fuzzier and not well defined, suggesting partial degradation of these complexes in these conditions. The protein composition of B2-B5 from G and O samples was subsequently investigated by SDS-PAGE (Fig. 4). B2 and B3 were characterized by four bands migrating at around 30 kDa, as expected for LHC antenna proteins. Interestingly, the two bands at lower molecular weight (MW) were more abundant in B2 than in B3 for both G and O samples, suggesting the preferential monomeric state of some specific LHC proteins as in the case of CP26 and CP29 in *C. reinhardtii* ^28,40^. In B4 fractions, PSII-core proteins such as CP43, CP47, D1, D2 and PsbO subunits were identified on the basis of their apparent MW^29^. In B5 fractions (PSI-LHCI), high MW bands (60–70 KDa) could be attributed to PsaA and PsaB, together with bands at low MW (<30 KDa) attributable to LHCI antenna proteins and other PSI core subunits. The pigment binding properties of the different isolated fractions were then analyzed by HPLC (Table 1). Pigment results from B2 and B3 fractions were normalized to 14 Chls, as previously reported for LHCII subunits from higher plants^41^. Chl a/Chl b ratios were similar in B2 and B3 fractions from G, O or R samples, while Chl/Car ratios were increased in O and R as compared to G. This was mainly due to a strong reduction in violaxanthin content which is likely related to reduced stability of the V1 site in the presence of zeaxanthin, as previously reported for LHC proteins isolated from higher plants^42,43^. Traces of zeaxanthin were indeed detected in B2 and B3 from O and R cells. Zeaxanthin accumulation at the V1 sites of the LHC protein is likely due to xanthophyll cycle activation during high light stress or zeaxanthin accumulation in O and R membranes as a precursor to astaxanthin^20,42,44,45^. The reduced stability of the V1 site in B2 and B3 complexes from O and R cells could also be the reason for the reduced content of lutein in these fractions as compared to B2 and B3 fractions from G samples. Since more than 2 luteins were found in B2 and B3 fractions, the extra lutein is likely bound to the peripheral site V1^46^, which however is partially empty in O and R samples. Astaxanthin was almost completely absent in LHC proteins, even if traces of this ketocarotenoid were present only in O/R B2 and B3 fractions. The possible affinity of LHCII complexes for astaxanthin has indeed been previously investigated by *in vitro* reconstitution^47^. B4 fractions were characterized by a high Chla/Chlb ratio (>20), as expected for PSII-core. Traces of Chl b, neoxanthin and violaxanthin were also detected, and are likely to arise from the residual presence of antenna proteins. The most evident difference between G and O of B4 fractions is a decrease in β-carotene which is partially replaced in O by astaxanthin. In particular ≈2.5 molecules of astaxanthin were bound by PSII-core in O, mainly in its esterified form. It is worth noting that violaxanthin and lutein were also found in decreased quantities in O B4 as compared to G B4. These xanthophylls are likely related to residual LHC proteins bound to B4, and it is not possible exclude a possible substitution of these pigment with astaxanthin in O B4. PSI-LHCI fractions (B5 fraction) were characterized by a Chl a/Chl b ratio of ≈7, an intermediate value between the Chl a/b ratio previously measured in the case of PSI-LHCI isolated from higher plants^31^ and *C. reinhardtii* ^48^. The lower Chl a/b ratio observed in *C. reinhardtii* is due to an increased content of Lhca proteins, with 7–9 Lhca subunits bound per reaction center as compared to the 4 Lhca subunits found in the case of *A. thaliana* ^26,32,48,49^. In order to estimate the Lhca content associated to the PSI reaction center in *H. pluvialis*, we assumed 3.4 Cars per Lhca subunit as previously reported in the case of *A. thaliana* and *C. reinhardtii* ^48^ and 100 Chl a molecules per PSI core complex^33,50–52^. From the Chl a/b and Chl/Car ratios of the B5 fractions, we estimated 5 Lhca proteins per P700, with 14 Chls and 3.4 Cars bound by each subunit and 170 Chls bound by the PSI-LHCI complex. An intermediate value of Lhca content per PSI-LHCI complex in *H. pluvialis* as compared to higher plants and *C. reinhardtii* was then confirmed by PSI-LHCI functional antenna size measurement on whole cells (Supplemental data Figure S2). Comparing B5 from G and O samples, a ~21% decrease in β-carotene content was evident, with a loss of ~2.9 molecules per P700. Conversely, ~2.8 astaxanthin molecules were found bound to each O B5 complex, suggesting a possible substitution of β-carotene with astaxanthin. A 28% decrease in violaxanthin was also observed in O B5 as compared to G B5, coupled with a rise in zeaxanthin, lutein and neoxanthin. A general re-organization of Car binding sites was thus evident in O samples even if the same total amount of Car was found in G or O B5 complexes. The markedly increased carotenogenesis observed in O cells leading to high accumulation of lutein, zeaxanthin and astaxanthin in thylakoid membranes is likely to be the reason for the different pigment binding properties of B4 and B5 complexes.

**Table 1:** Pigment analysis of isolated photosynthetic complexes. Pigment extracts were analyzed by HPLC. Pigment data from B2 and B3 fractions were normalized to 14 chlorophylls; pigments from B4 fractions were normalized to 72 chlorophylls; pigments from B5 fractions were normalized to 170 chlorophylls. Chl: chlorophyll; Car: carotenoid; Neo: neoxanthin; Viola: violaxanthin; Antera: anteraxanthin; Lute: Lutein; Zea: zeaxanthin; β-Car: β-carotene; Asta: astaxanthin; Asta ester: esterified forms of astaxanthin. Standard deviations are below 8% for each value reported in the table (n = 3).

	Chl	Chla/Chlb	Chl a	Chl b	Chl/Car	Neo	Viola	Asta	Antera	Lute	Zea	Asta ester	β-Car
GREEN B1	100	2.8	73.7	26.3	0.8	6.9	36.9	n.d.	n.d.	73.8	n.d.	n.d.	6.2
ORANGE B1	100	3.3	76.9	23.2	0.2	11.5	59.6	10.2	11.8	69.6	8.0	434.6	6.6
RED B1	100	4.2	80.9	19.1	0.1	12.0	105.2	17.8	23.1	91.4	14.7	1352.5	27.3
GREEN B2	14	1.6	8.6	5.4	4.0	0.7	0.7	n.d.	n.d.	2.7	n.d.	n.d.	n.d.
ORANGE B2	14	1.5	8.4	5.6	4.7	0.9	0.2	<0.1	n.d.	2.3	0.1	<0.1	<0.1
RED B2	14	1.5	8.4	5.6	4.5	0.9	0.2	<0.1	n.d.	2.4	0.1	0.1	<0.1
GREEN B3	14	1.7	8.8	5.2	3.8	0.7	0.8	n.d.	n.d.	2.8	n.d.	n.d.	n.d
ORANGE B3	14	1.5	8.5	5.5	4.8	0.8	0.3	<0.1	n.d.	2.3	0.1	n.d.	n.d.
RED B3	14	1.5	8.4	5.7	4.4	0.9	0.2	0.1	n.d.	2.3	0.1	0.1	n.d.
GREEN B4	72	22.6	68.9	3.1	4.3	0.3	1.0	n.d.	n.d.	3.3	n.d.	n.d.	12.1
ORANGE B4	72	29.4	69.6	2.4	6.3	0.6	0.6	0.6	n.d.	2.2	n.d.	1.9	5.4
GREEN B5	170	7.1	149.1	20.9	4.4	0.2	8.2	n.d.	n.d.	17.0	n.d.	n.d.	13.5
ORANGE B5	170	7.0	148.7	21.3	4.4	0.8	6.0	1.9	n.d.	18.0	1.0	0.9	10.6

### Excitation energy transfer in astaxanthin binding complexes

**Figure 5:**
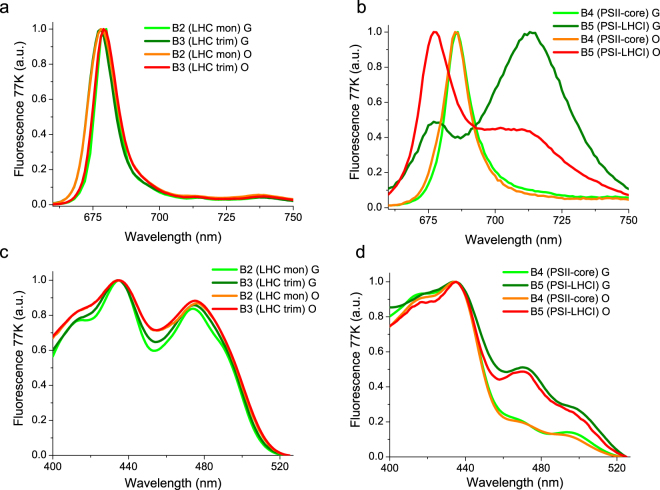
77 K Fluorescence emission and excitation spectra of isolated pigment binding complexes. Panel a/b:77 K fluorescence emission spectra of B2-3 (Panel a) and B4-5 (Panel b) fractions, upon excitation at 440 nm. Panel c/d: 77 K fluoresce excitation spectra of B2-3 (Panel c) and B4-5 (Panel d) fractions. Emission wavelengths were set at 680 nm for B2 and B3, 687 for B4 and 715 for B5 fractions.

The functional properties of astaxanthin bound to photosynthetic complexes were initially investigated by fluorescence measurements at 77 K, where emission is mainly attributed to the lowest Chl excited states. When exciting Chl a at 440 nm, B2 and B3 fractions from G cells showed similar emission peaks at 680 nm and similar excitation spectra characterized by a high Chl b contribution (Fig. 5). The traces of astaxanthin found in LHC proteins (Table 1) do not influence the fluorescence properties of these fractions. PSII-core fractions (B4) from both G and O samples were characterized by an emission spectrum peaking at 686 nm and an excitation spectrum almost absent of Chl b contribution, as expected for a PSII-core. Even in this case, astaxanthin binding to PSII core did not influence the fluorescence properties of the complex. PSI-LHCI complex (B5) isolated from G cells presented two separated peaks; a major peak at 715 nm related to emission from the low energy Chls bound to the complex, and a minor peak at 680 nm associated to partially dissociated antenna proteins. In PSI-LHCI from O cells, the 680 nm emission peak was more dominant than the 715 nm peak, suggesting a higher proportion of detached LHCI subunits. The excitation spectra of the 715 nm emission peaks associated to the intact PSI-LHCI complex were similar for both G and O samples. Astaxanthin binding to the different complexes does not alter the energy of the emitting state, but could be involved in a partial disconnection of LHC proteins from PSI core complex.

**Figure 6:**
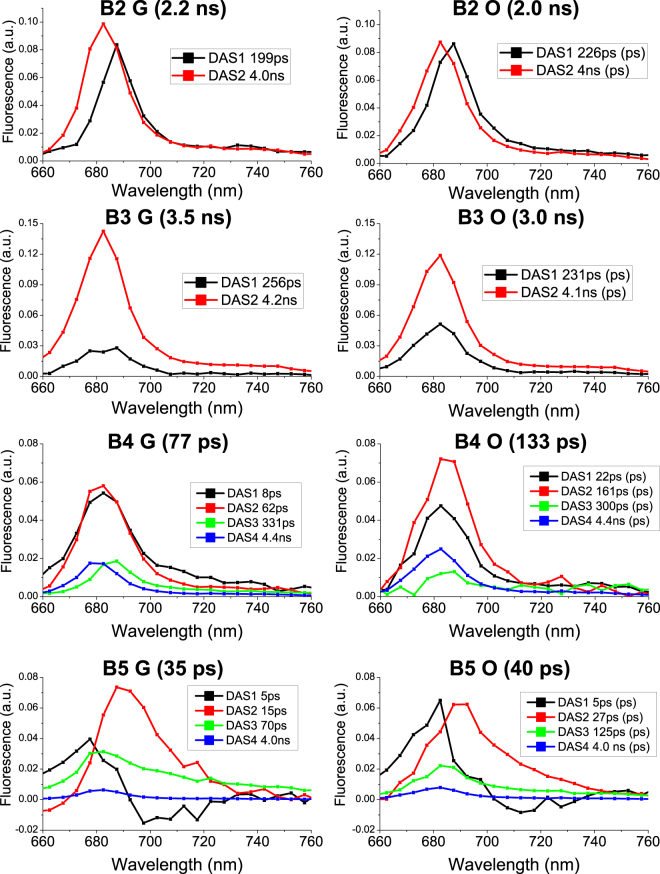
Decay Associated Spectra (DAS) resulting from Global Analysis of fluorescence decay maps of isolated pigment binding complexes. 2D Streak camera maps were fitted with a multi-exponential decay function with a Global Analysis approach. The resulting wavelength dependent amplitudes, ${A}_{i}(\lambda )$, are referred to as Decay Associated Spectra (DAS), with each DAS being associated with an exponential decay constant, ${\tau }_{i}$. Decay Associated Spectra (DAS) of each sample are reported with associated decay constants indicated in the legend. Average fluorescence lifetimes for each sample is reported in brackets and calculated as ΣA_i_τ_i_/ΣA_i_. Two exponential decay components were required to adequately fit the decay maps recorded for B2 and B3, whilst four were required for B4 and B5.

Excitation energy transfer dynamics were subsequently investigated by time resolved fluorescence spectroscopy with a streak camera based set up. Streak camera detection allows simultaneous acquisition of fluorescence decays at different wavelengths. The resulting datasets were analyzed by global analysis, resulting in decay associated spectra (DAS) for each sample – wavelength dependent amplitudes for each time-constant in a multi-exponential decay^53^. DAS identified in each sample were normalized to the total DAS amplitude of that sample. As reported in Fig. 6, two components were sufficient to fit B2 and B3 decays, with a shorter redder component at ~200 ps (DAS1_B2/3_) and a longer bluer component at ~4 ns (DAS2_B2/3_). DAS1_B2/3_ and DAS2_B2/3_ could be assigned to two different LHC protein conformations with different non photochemical quenching properties, as previously reported^54^. DAS1 was higher in B2 than in B3 fractions and stronger in O than in G samples, indicating stronger quenching properties in B2 and in O samples. Accordingly, average fluorescence decay times (τ_AV_) were shorter in B2 than in B3 fractions and in O than in G samples (Fig. 6). The results obtained in the case of B2 and B3 are consistent with fluorescence decay kinetics of LHC monomers and LHCII trimers respectively^54,55^. The reduced τ_AV_ observed in the case of O fractions is unlikely to be related to the traces of astaxanthin or zeaxanthin bound to the complexes, but rather to varying protein compositions of these fractions induced by the different growth conditions and accumulation of protein subunits with stronger quenching properties^54^. In the case of B4 fractions, four DAS were identified. Differences were found mainly in the first two fast decaying DAS1_B4_ and DAS2_B4_, with decay constants of 8/22 ps, and 62/161 ps for Green/Orange B4 respectively. DAS1_B4_ and DAS2_B4_ amplitudes were similar in G samples, while in O samples DAS2_B4_ had an increased weight as compared to DAS1_B4_. DAS3_B4_ and DAS4_B4_ on the other hand exhibited similar time constants (300 ps and 4.4 ns respectively) in both G and O samples, with DAS4_B4_ being more represented in O samples. DAS1-4_B4_ identified for B4 are consistent with components previously reported for PSII core complexes, even if it is difficult to associate components unambiguously to Chl or protein moieties^56^. Only the weak 4.4 ns component (DAS4_B4_), most clearly visible in the O sample, can be safely associated to partially detached antenna proteins or free Chls found in B4. A 77 ps τ_AV_ was calculated for B4 isolated from G cells, consistent with previous reports for PSII core^27^. The longer τ_AV_ determined in the case of B4 isolated from O cells (136 ps) suggests that excitation energy transfer to the PSII reaction center is partially disturbed in this complex. Four DAS components were also identified for the PSI-LHCI complexes (B5). In particular a short (5 ps) component, DAS1_B5_, with positive/negative amplitude was found in both G and O PSI-LHCI complexes and are attributed to energy equilibration within the complex^57^. The 13 ps DAS2_B5_ found in B5 from G samples has been usually associated to emission from PSI-core. In the case of B5 from O cells, DAS2_B5_ is characterized by time constant of 27 ps which is longer than that in G samples and thus indicates an alteration of excitation energy transfer to the reaction center. DAS3_B5_ found in the B5 fraction from G cells has a time constant of 70 ps and a spectrum which is more enriched in forms emitting above 700 nm. This component is related to energy transfer from peripheral LHCI complexes. In PSI-LHCI from O cells, the time constant associated with DAS3_B5_ is increased to 125 ps whilst the DAS amplitude is reduced. This indicated some alterations in energy transfer from antenna complexes to the PSI reaction center. Finally, a small 4 ns component (DAS4_B5_) was identified in both B5 fractions from G and O cells, and was attributed to detached antenna proteins as previously observed in PSI-LHCI preparations^31,44,48,57,58^. This ns component was almost 50% stronger in PSI-LHCI from O cells. Astaxanthin binding PSI-LHCI complexes from O cells were thus characterized by reduced excitation energy transfer to the reaction center from both the Chl moieties bound to the core complex and to the peripheral antenna proteins. They also contain a higher amount of partially disconnected LHCI proteins emitting in the ns time range, in agreement with the low temperature emission florescence spectra reported in Fig. 5. In the case of PSI-LHCI, the photochemical efficiency (ϕPSI) of the complex can be estimated from the τ_AV_ (35 and 40 ps respectively for G and O samples), which in turn can be interpreted as the time required to transfer energy to the reaction center of the complex. ϕPSI calculated from PSI-LHCI τ_AV_ and disregarding the ns component (as previously reported^57^) were in both cases higher than 98%. Inclusion of the ns component reduced the excitation energy transfer efficiency to 92% and 95% for the O and G samples respectively. Astaxanthin binding PSI-LHCI is thus characterized by a partial disconnection of LHCI proteins, whilst maintaining more than 90% of excitation energy transfer efficiency, as previously observed for PSI-LHCI complexes^31,48,59^.

### Photoprotective functions of astaxanthin in the plastids

**Figure 7:**
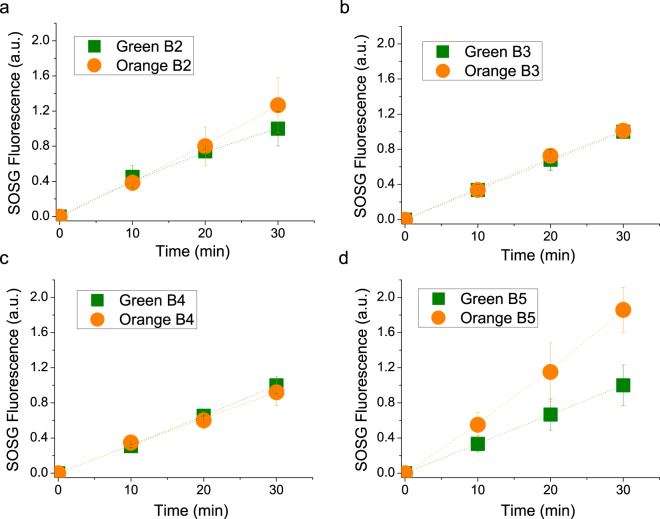
Singlet oxygen production in isolated complexes upon red light treatment. Singlet oxygen production was indirectly determined following the increase of fluorescence of Singlet Oxygen Sensor Green (SOSG), a fluorescent probe increasing its fluorescence in presence of singlet oxygen. Isolated complexes were illuminated with red light at 2000 µmol m^−2^s^−1^. All data were normalized to chlorophyll content and to the SOSG fluorescence of the green samples after 40′ of illumination. Standard deviations are indicated in each panel (n = 3).

**Figure 8:**
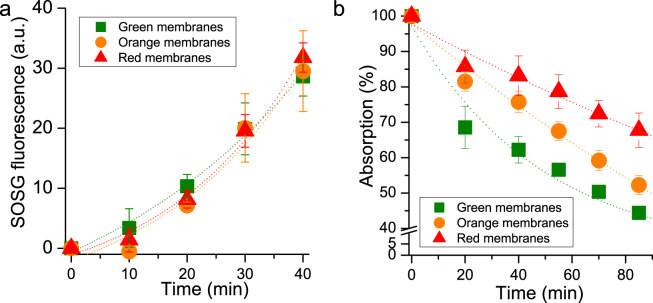
Singlet oxygen production and chlorophyll photo-bleaching in isolated membranes. Panel a: singlet oxygen production indirectly determined following the increase of fluorescence of Singlet Oxygen Sensor Green (SOSG), a fluorescent probe increasing its fluorescence in presence of singlet oxygen. Isolated membranes were illuminated with red light at 6000 µmol m^−2^s^−1^. All data were normalized to chlorophyll content and to the SOSG fluorescence of green membranes after 30′ of illumination. Panel b: chlorophylls photo-bleaching induced in isolated membranes upon illumination with white light at 6000 µmol m^−2^s^−1^. Standard deviations are indicated in both panels (n = 3).

The photoprotective role of astaxanthin bound to Chl binding subunits was evaluated by measuring ^1^O_2_ production under high irradiance (2000 µmol m^−2^s^−1^) of red light (>600 nm) and application of a fluorescent probe (Singlet Oxygen Sensor Green, SOSG) whose fluorescence increases upon ^1^O_2_ production^60^. The use of red light, which is absorbed only by Chls, enables selective investigation of the photoprotective role of astaxanthin without regard to its absorption properties^61,62^. As reported in Fig. 7, after 30 minutes of illumination no significant differences were observed among G and O B2, B3 and B4 fractions (Fig. 7a–c). In the case of PSI-LHCI complexes, O B5 showed a higher ^1^O_2_ production than G B5 (Fig. 7d). This can be explained by the presence of some antenna proteins in O B5 which transfer excitation energy less efficiently to the PSI reaction center, in agreement with 77 K steady state and time resolved fluorescence results: these antenna proteins are more prone to produce ROS upon high light illumination. In every case tested, no significant improvements in photoprotection were attributable to astaxanthin binding. Since most of the astaxanthin was found not bound to Photosystems, the same SOSG analysis was performed on isolated membranes illuminated with red light at 6000 µmol m^−2^s^−1^ for 40 minutes (Fig. 8a). Despite the huge amount of astaxanthin in O and R membranes, ^1^O_2_ production comparable with G thylakoids was found after normalization to Chl content, suggesting a minor role of astaxanthin as scavenger of ^1^O_2_. Moreover, astaxanthin in plastids could act as a light filter: in order to investigate this point, Chl bleaching was measured in these membranes upon white light treatment at 6000 µmol m^−2^s^−1^. As reported in Fig. 8b, Chl absorption in G thylakoids was reduced by 60% after 80 minutes due to Chl degradation, while the O and R Chl bleaching kinetics were much slower, especially in the case of R samples. Astaxanthin thus improves photoprotection in thylakoid membranes only through its absorption properties, acting as a light filter.

## Discussion

In this work, we presented the biochemical and spectroscopic properties of photosynthetic complexes responsible for light harvesting and energy conversion in *H. pluvialis*. In the case of cells grown in control conditions (G), monomeric and trimeric LHC proteins isolated from *H. pluvialis* present features consistent with previous report for LHC proteins purified from *A. thaliana* or *C. reinhardtii*. Interestingly, monomeric LHC proteins from *H. pluvialis* were characterized by a shorter fluorescence lifetime as compared to LHCII trimers. This finding is likely related to a somewhat different protein composition of B2 fractions as compared to B3 (Fig. 4), with B2 containing some LHC proteins more abundant in monomeric form, such as the minor monomeric LHC subunits identified in *A. thaliana* (Lhcb4-6 subunits) and *C. reinhardtii* (CP26 and CP29)^27,54^. PSII core complexes were also isolated and analyzed, demonstrating conservation of pigment binding properties and similar biochemical and spectroscopic properties as compared to PSII cores purified from cyanobacteria or higher plants. The evolution of the photosynthetic process therefore mainly addressed the peripheral light harvesting complexes rather than core complexes^27,31^. In the case of PSI-LHCI, 77 K fluorescence demonstrates that *H. pluvialis* lacks Lhca proteins with so called “red-forms” which emit above 730 nm and are found in higher plants but not in green algae^31,48^. It is postulated that the absence of far red light in water did not lead microalgae to evolve LHC proteins which absorb above 700 nm. Chl a/b and Chl/Car ratios measured in the case of B5 fractions suggest the presence of 5 Lhca proteins per reaction center, however additional biochemical and structural work is required to support this hypothesis. Nevertheless, the PSI functional antenna size measured following P700 oxidation kinetics indicated an intermediate value between *A. thaliana* (4 Lhca per P700)^26^ and *C. reinhardtii* (7/9 Lhca per P700)^32,48^ (Supplemental data Figure S2). *H. pluvialis* PSI-LHCI was characterized by a very short τ_AV_ (<40 ps), indicating a ϕPSI higher than 98% and consistent with similar analysis performed on PSI-LHCI complexes purified from other organisms^48,57^. The photosynthetic machinery is reorganized when *H. pluvialis* cells are stressed and astaxanthin is accumulated. Oxidative stress and ROS accumulation are indeed the triggers for activation of the astaxanthin biosynthetic pathway, with β-carotene over-production in the chloroplast followed by export to the cytosol and conversion to astaxanthin. In particular, while the different enzymes involved in β-carotene accumulation are localized in the plastids, the key enzyme β-carotene oxygenase (CRTO), which produces astaxanthin from β-carotene or zeaxanthin, was found both in the plastid and in lipid vesicles in the cytosol, despite its activity only having been previously reported in the cytosol compartment^20^. Our findings of traces of astaxanthin in the plastids suggests that a low activity of CRTO was present also in these organelles. The photosynthetic machinery continues to work even in cysts^12^, but the chloroplasts reduce their volume and the thylakoid membranes become degraded^25,63^. In this work, we demonstrated that acclimation to high light induced a destabilization of the PSI-LHCI supercomplex and PSII core, especially after six days of high irradiance (Fig. 1). Rapid turnover of PSII core subunits is likely to be the reason for the rapid destabilization observed for the PSII core. In addition, isolated PSII cores from O cells were characterized by a slower excitation energy transfer to the reaction center. In the PSII core complex, β-carotene molecules are in close contact with Chls and are required for effective quenching of ^3^Chl* and scavenging of ^1^O_2_ produced during charge recombination. Therefore, depletion of β-carotene produces a strong photooxidation in both PSII and PSI core complexes^64^. In the case of PSI-LHCI, high light acclimation caused a destabilization of the interaction between peripheral antenna complexes and PSI core, as demonstrated by both 77 K steady state and time resolved fluorescence. Moreover, the reduced energy connection between antenna proteins and PSI reaction center decreased the photochemical quenching of the LHCI proteins, exposing them to a higher risk of photooxidation, as measured using SOSG (Fig. 7). The molecular mechanism by which PSII core and PSI-LHCI are destabilized cannot be easily identified and additional work is required to elucidate this point. The loss and partial substitution of β-carotene with astaxanthin in PSII core and PSI-LHCI could however be involved in the destabilization of Photosystems. Astaxanthin binding to Photosystems I been reported for Chlrophyceae species such as Eremosphaera viridis^65^, however no information was available for the main species used in astaxanthin production, *H. pluvialis*. *H. pluvialis* astaxanthin binding complexes were not more photoprotected as compared to the control samples and their excitation energy transfer dynamics were even slower when compared to the same complexes isolated in the absence of astaxanthin. It is thus difficult to claim that astaxanthin binding to PSI or PSII has a photoprotective role. However, considering the higher level of ^1^O_2_ produced in astaxanthin binding PSI-LHCI and the similar ^1^O_2_ production observed in isolated membranes, it cannot be excluded that astaxanthin found free in the thylakoid membranes could have a role as scavenger of ^1^O_2_ produced by Photosystems. Rather, considering the important role of β-carotene for the assembly and function of PSI and PSII^33,66^, its substitution by astaxanthin could be the key to core complex destabilization. Indeed, astaxanthin was found in PSI and PSII cores even in its esterified form. Interactions between the fatty acids esterified to astaxanthin and the protein subunits of photosystems could impact the interactions at the base of the PSI and PSII assemblies. These results are indeed consistent with the observation of reduced photochemical efficiency in higher plants engineered to accumulate astaxanthin^36,38,67^. Considering the ratio between astaxanthin and Chls in whole cells and in isolated fractions, less than 1% of the total astaxanthin accumulated in *H. pluvialis* was found bound to PSI or PSII, while almost all astaxanthin is accumulated in the cytoplasm. Astaxanthin rich oil droplets accumulated in the cytoplasm could have a specific role as antioxidants to protect the nucleus. Moreover, the astaxanthin oil droplets act as a light filter, reducing the excitation pressure on photosynthetic subunits and their risk of photodamage (Fig. 8b)^12^. The presence of astaxanthin in *H. pluvialis*, even in photosynthetic pigment binding complexes, raises the question whether these astaxanthin molecules are synthetized in the plastid or, perhaps more likely, in the cytoplasm and then imported back to the plastid.

## Methods

### Strain and culture conditions

*Haematococcus pluvialis* strain K-0084 was obtained from Scandinavian Culture Collection of Algae & Protozoa. Liquid cultures were grown photoautotrophically at 40 µmol photons m^−2^s^−1^ on BG-11 medium at 22 °C in flasks^12^. Culture mixing was provided by bubbling filtered (0, 2 µm) air. High light treatment at 400 µmol photons m^−2^s^−1^ was applied to cell cultures in their exponential phase (approximately 5 × 10^5^ cells ml^−1^). Each experiment was repeated in at least five independent experiments with three biological replicates for each sample.

### Cell concentration and pigment analysis

Cell concentrations (cells mL^−1^) were determined manually using a Neubauer counting chamber as described in^12^. Pigment analysis were performed by reverse phase HPLC as described in^68^. In particular, pigment extracts in acetone 80% were analyzed by Thermo-Fisher HPLC system equipped with a C18 column using a 15-min gradient of ethyl acetate (0 to 100%) in acetonitrile-water-triethylamine (9:1:0.01, vol/vol/vol) at a flow rate of 1.5 ml/min. Only in the case of whole cells pigmentation extraction was performed in DMSO as described in^12^. Pigment detection was done by a Thermo-Fisher 350–750 nm diode array detector.

### Thylakoid membranes and pigment binding complexes isolation

Thylakoid membranes were isolated from *H. pluvialis* cells as described in^69^, with some modifications. *H. pluvialis* cells, were harvested by centrifugation (1500 g, 3 min) and resuspended in B1 buffer (50 mM tricine pH 7.9, 0.35 M sorbitol, 10 mM NaCl, 5 mM MgCl_2_, 0.5% dried powdered milk, 1 mM aminocaproic acid, 0.2 mM aminobenzamidine, and 0.2 mM phenylmethylsulfonyl fluoride) at a final concentration of 10^6^ cells/ml and then passed through a prechilled (4 °C) Cell-disrupter (Constant Systems, Northants, UK) at 2.5 kbar. The resulting homogenate was subsequently centrifuged at 1500 g for 3 min at 4 °C, to remove intact cells. The supernatant was collected and centrifuged at 12000 g for 15 minutes at 4 °C. The resulting thylakoid membrane pellet was resuspended in B2 buffer (20 mM tricina pH 7.9, 50% glycerol, 10 mM NaCl, 5 mM MgCl_2_, 1 mM aminocaproic acid, 0.2 mM aminobenzamidine, and 0.2 mM phenylmethylsulfonyl fluoride) and immediately used for analysis, or stored at −80 °C, after freezing it in liquid nitrogen. Isolated thylakoids were cleaned by ultracentrifugation in a sucrose step gradient formed by 1.9 M, 1.3 M and 1.14 M sucrose, 25 mM Hepes pH 7.0 and 10 mM EDTA. Clean thylakoid membranes were recovered from the 1.3 M layer, diluted to reduced sucrose concentration and precipitated by centrifugation. Isolated thylakoids were then solubilized at a concentration of 1 mg/ml of Chls (200 µg of Chls in total), with β-DM 1% and loaded onto a sucrose gradient (0.1–1 M) in presence of 0.06% β-DM and 10 mM Hepes pH 7.5. Protein fractions were isolated upon ultracentrifugation, collected from sucrose gradient and then cleaned by anion exchange chromatography as described in^70^. Anion exchange chromatography was performed on TOYOPEARL DEAE-650S resin (Sigma-Aldritch) equilibrated with 0.06% β-DM and 10 mM Hepes pH 7.5: protein elution was achieved using an elution buffer composed by 0.5 M NaCl, 0.06% β-DM and 10 mM Hepes pH 7.5.

### Absorption and fluorescence spectroscopy

Absorption spectra were measured by DW2000 Aminco spectrophotometer as described in^71^. 77 K steady state emission spectra were recorded using a Fluoromax3 equipped with an optical fiber (Horiba Jobin Yvon) as described in^39^. Emission spectra were performed by exciting the sample at 440 nm with an excitation bandwidth of 5 nm and recording emission in the 650–800-nm range (emission bandwidth of 1 nm). Excitation spectra at 77 K were performed upon excitation in the 400–550 nm range (excitation bandwidth of 2 nm) measuring the fluorescence emitted at 680 or 715 nm (emission bandwidth of 3 nm) as described in the text.

### SDS-PAGE analysis

Denaturing SDS-PAGE was performed with Tris-Tricine buffer systems^72^.

#### PSI functional antenna size

Relative PSI antenna size was estimated from kinetics of P700 oxidation in limiting orange light (12 μE m^−2^ s^−1^) in whole cells treated with DCMU (3-(3,4-dichlorophenyl)−1,1-dimethylurea), DBMIB (2,5-dibromo-3-methyl-6-isopropylbenzoquinone), ascorbate and methyl-viologen, as described in^73^.

### Time resolved fluorescence measurements

Time-resolved fluorescence measurements were performed using a femtosecond laser excitation at 440 nm and a streak camera detection system, as reported in^44^. Briefly, an unamplifed Ti:sapphire laser (Coherent Chameleon Ultra II) operating at 80 MHz was tuned to provide pulses with central wavelengths of 880 nm, energies of 30 nJ, and temporal and spectral bandwidths of 140 fs and 5 nm, respectively. A β-barium borate crystal provided type I phase-matched second harmonic generation, leading to pulses with central wavelengths of 440 nm. These were focused onto the sample, maintaining a low fluence (<30 mJ/cm^2^, 100 mm spot diameter) in order to avoid saturation and degradation effects in the sample. The samples were kept at a constant temperature of 11 °C by a temperature controlled cuvette cooled by a peltier system. The resulting collected emission was analyzed by a spectrograph (Princeton Instruments Acton SP2300) coupled to a streak camera (Hamamatsu C5680) equipped with a synchroscan voltage sweep module. In this way, measurements of photoluminescence intensity as a function of both wavelength and time were obtained with spectral and temporal resolutions of ~1 nm and ~3 ps respectively. Temporal broadening of the pump pulses caused by dispersive elements was confirmed to be well below the response time of the detection system.

### Global analysis

Streak camera fluorescence decay maps were globally fitted with exponential functions as previously reported^44,53^. Briefly, the experimental datasets were fitted using a multi-exponential function as described by equation (1)

1 $$I(\lambda ,t)={\sum }_{i=1}^{n}{A}_{i}(\lambda ){e}^{-t/{\tau }_{i}}$$

with $I(\lambda ,t)$ the wavelength- and time-resolved fluorescence intensity, and ${A}_{i}$ the amplitude of the exponential decay ${e}^{-t/{\tau }_{i}}$. Whilst the amplitudes were treated as wavelength dependent (${A}_{i}={A}_{i}(\lambda )$), the exponential decay constants were assumed to be wavelength independent ${\tau }_{i}\ne {\tau }_{i}(\lambda )$. The resulting wavelength dependent amplitudes, ${A}_{i}(\lambda )$, are referred to as Decay Associated Spectra (DAS), with each DAS being associated with a particular exponential decay constant, ${\tau }_{i}$. It is important to note that DAS are simply parameterisations of a time-resolved fluorescence dataset in a multi-exponential temporal basis, and so most often cannot be assigned a physical origin.

Average fluorescence lifetimes were calculated as described by equation (2):

2 $${\tau }_{AV}={\sum }_{i=1}^{n}{A}_{i}{\tau }_{i}/{\sum }_{i=1}^{n}{A}_{i}\,$$

where ${A}_{i}$ is the spectrally integrated amplitude over the spectral range 650–780 nm.

### Singlet oxygen production

Singlet oxygen production was measured *in vivo* by following the 532 nm fluorescence emission of a singlet oxygen sensor green probe^60^. In particular, samples were diluted to in order to reach the same maximum at 0.15 OD in the Qy region and Singlet Oxygen Sensor Green was added to a final concentration of 5 μM. Samples were then illuminated with red light (2000 μE m^−2^ s^−1^ in the case of isolated complexes, 6000 μE m^−2^ s^−1^ in the case of thylakoids) and a regular time intervals, fluorescence at 532 nm was registered. Data were analyzed as increase in percentage of fluorescence, compared to time 0. Experimental data were then fitted with exponential functions.

### Data Availability

All the data generated during and/or analyzed during the current study are available from the corresponding author on reasonable request.

## Electronic supplementary material

Supplementary data Figure S1-2
