# Supplementary material for: Functional analysis of photosynthetic pigment binding complexes in the green alga Haematococcus pluvialis reveals distribution of astaxanthin in Photosystems
Source: Sci Rep. 2017 Nov 24;7:16319. doi: 10.1038/s41598-017-16641-6 (PMC5701160; doi:10.1038/s41598-017-16641-6)

Functional analysis of photosynthetic pigment binding complexes in the green alga *Haematococcus pluvialis* reveals distribution of astaxanthin in Photosystems

Francesco Mascia[1+], Laura Girolomoni[1+], Marcelo J. P. Alcocer[2], Ilaria Bargigia[2], Federico Perozeni[1], Stefano Cazzaniga[1], Giulio Cerullo[3], Cosimo D'Andrea[2,3], Matteo Ballottari[1*]

[1] Dipartimento di Biotecnologie, Università di Verona, Strada Le Grazie 15, I-37134 Verona, Italy

[2] Center for Nano Science and Technology @PoliMi, Istituto Italiano di Tecnologia, via Pascoli 70/3, 20133 Milano, Italy

[3] IFN-CNR, Department of Physics, Politecnico di Milano, P.za L. da Vinci 32, 20133 Milano, Italy

[+]Authors contributed equally to the work

[*]Corresponding author: matteo.ballottari@univr.it

SUPPLEMENTAL DATA

Figure S1. Absorption spectra of B1 fraction isolated from *H. pluvialis*. Absorption spectra of B1, fractions (Figure 1d) were normalized to the maximum absorption peak in the 600 nm and 700 nm region.

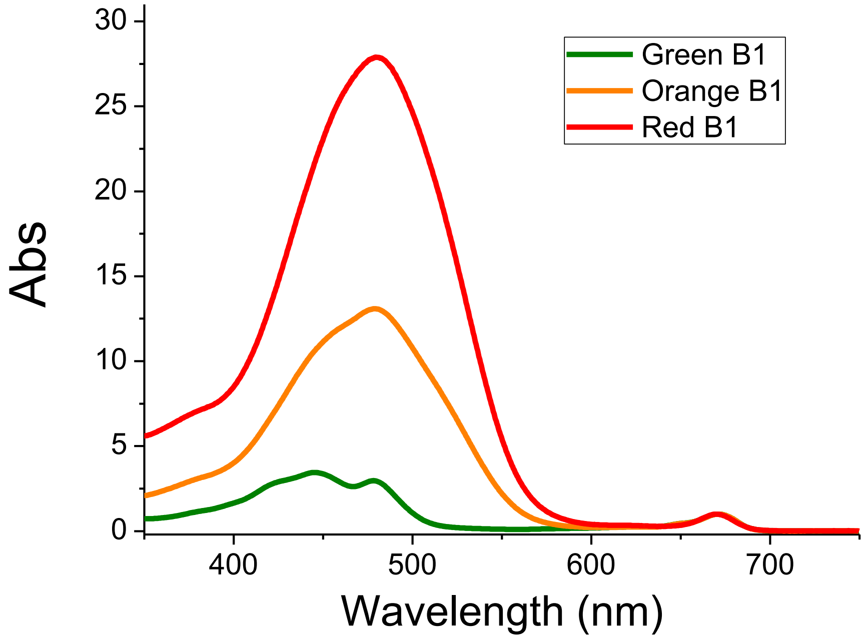

Figure S2. Functional antenna size of Photosystem I in *H. pluvialis* compared to *Arabidopsis thaliana* and *Chlamydomonas reinhardtii*. Panel a: P700 oxidation kinetics measured on whole cells in presence of DCMU and DBMIB, in order to block linear and cyclic electron transfer, and ascorbate and methyl viologen as electron donor and acceptor. Measurements were performed at 12 μmol $m^{-2}s^{-1}$. Traces representative of 5 independent biological replicates are reported. Panel b: functional antenna size of PSI-LHCI calculated as $1/\tau_{2/3}$, where $\tau_{2/3}$ is the time required to reach 2/3 of the maximum P700 oxidation. A.t.: *Arabidopsis thaliana;* C.r.: *Chlamydomonas reinhardtii*. H.p. *G/O: H. pluvialis* in Green/Orange stage as in Figure 1 main text. Standard deviations are indicated (n=5).

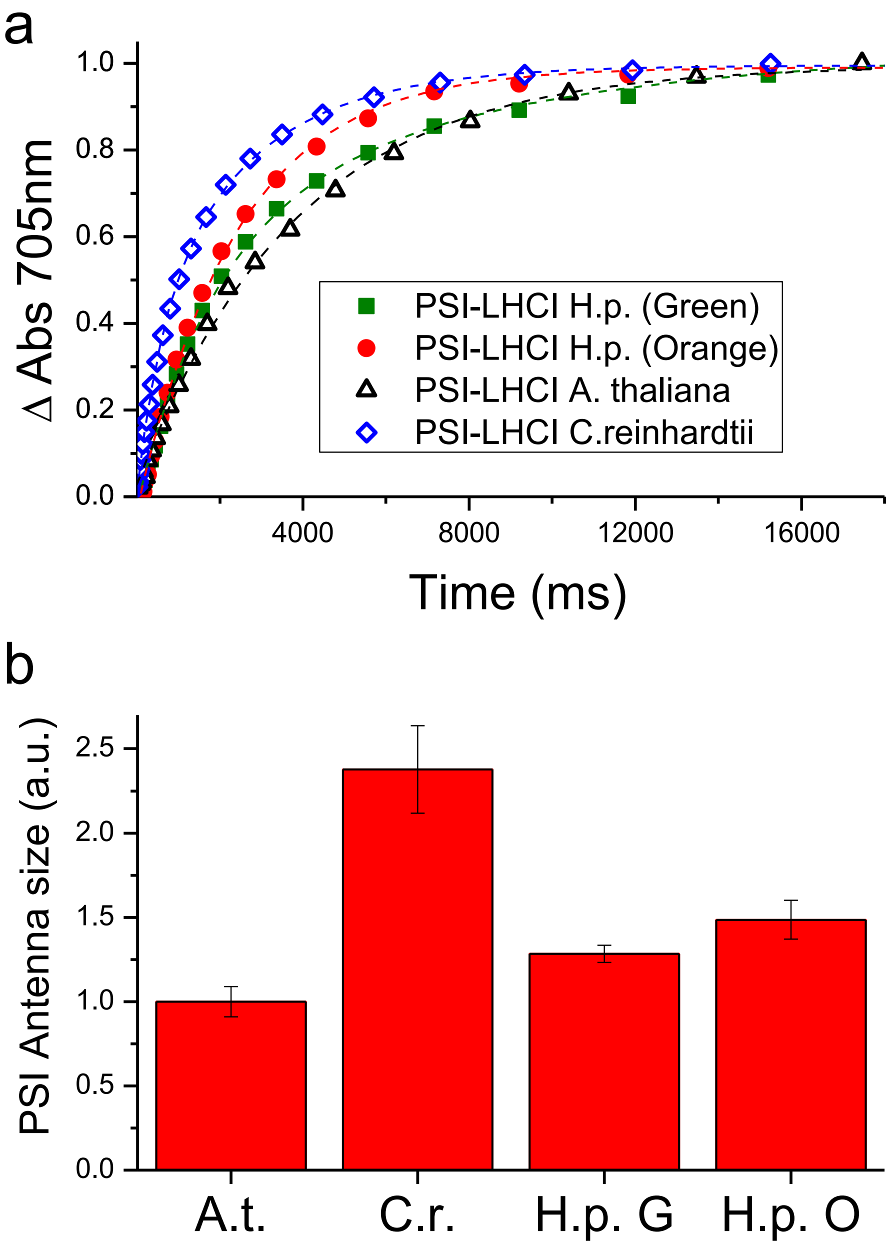

Supplement: Supplementary file 1 — Supplementary data Figure S1-2 [file 41598_2017_16641_MOESM1_ESM.pdf]
